# A Potential Role of the Translation Elongation Factor *eef1a1* in Gonadal High-Temperature Perception in Chinese Tongue Sole (*Cynoglossus semilaevis*)

**DOI:** 10.3390/ani12131603

**Published:** 2022-06-21

**Authors:** Qian Wang, Qian Liu, Wenxiu Ma, Rui Wang, Shuo Li, Zhongdian Dong, Changwei Shao

**Affiliations:** 1 Guangdong South China Sea Key Laboratory of Aquaculture for Aquatic Economic Animals, Fisheries College, Guangdong Ocean University, Zhanjiang 524000, China; wangqian2014@ysfri.ac.cn; 2 Laboratory for Marine Fisheries Science and Food Production Processes, Pilot National Laboratory for Marine Science and Technology, Yellow Sea Fisheries Research Institute, Chinese Academy of Fishery Sciences (CAFS), Qingdao 266071, China; liuqian97927@163.com (Q.L.); mawenxiu121@163.com (W.M.); abcwangrui2012@163.com (R.W.); shiyeyishang@outlook.com (S.L.); 3 College of Fisheries and Life Science, Shanghai Ocean University, Shanghai 201306, China; 4 Shandong Provincial Key Laboratory of Resistance Biology, College of Life Sciences, Shandong Normal University, Jinan 250014, China; 5 School of Marine Sciences, Ningbo University, Ningbo 315211, China; 6 Guangdong Provincial Key Laboratory of Pathogenic Biology and Epidemiology for Aquatic Economic Animals, Fisheries College, Guangdong Ocean University, Zhanjiang 524000, China

**Keywords:** *eef1a1*, high temperature, sexual response, sex differentiation, gonad, *Cynoglossus semilaevis*

## Abstract

**Simple Summary:**

The eukaryotic elongation factor 1 alpha (*eef1a*) gene is vital for protein translation by delivering aminoacylated tRNAs to the A/P site of the ribosome via the GTP-dependent reaction. Here, the Chinese tongue sole (*Cynoglossus semilaevis*) *eef1a1* gene was identified, and its potential role in gonadal high-temperature perception was assessed. The full-length sequence of *eef1a1* cDNA was 1809 base pair (bp) encoding a putative protein of 461 amino acids. The expression levels of *eef1a1* in the ovary were significantly higher than that in the testis from 6 mpf to 3 ypf. Under high-temperature induction during sex differentiation, *eef1a1* was significantly down-regulated in males, while the difference was not detected in females. Furthermore, the rapid response of *eef1a1* to environmental high temperature was assessed in vitro. Our findings suggest that *C. semilaevis eef1a1* might be essential for the molecular response regulatory network of external temperature affecting internal sex differentiation.

**Abstract:**

The eukaryotic translation elongation factor 1 alpha (*eef1a*) gene has a well-defined role in protein synthesis. However, its role in external temperature perception and internal sex differentiation and development is still unclear. In this study, *eef1a1* was identified and functionally analyzed in Chinese tongue sole (*Cynoglossus semilaevis*). The *eef1a1* cDNA, 1809 bp in length, had a 1386 bp open reading frame (ORF) that encoded a 461 amino acid polypeptide containing one EF-1_alpha domain. *eef1a1* expression levels were investigated across different tissues and during gonadal development. In the gonad, *eef1a1* showed a sexually dimorphic expression pattern with a statistically higher expression level in the ovary than in the testis from 6 months postfertilization to 3 years postfertilization. Under high temperature (28 °C) treatment during *C. semilaevis* sex differentiation (from 30 days postfertilization to 3 months postfertilization), *eef1a1* was statistically down-regulated in males, while the difference was not detected in females. In addition, the dual-luciferase assay exhibited that *eef1a1* can respond to high temperature rapidly. Based on these results, *C. semilaevis eef1a1* might have a dual role in the perception of external temperature changes and sex differentiation regulation.

## 1. Introduction

Sex determination and gonadal development is one of the key topics in developmental biology. In vertebrates, there are generally two main types of sex-determining mechanisms, genetic sex determination (GSD) and environmental sex determination (ESD) [1]. In species with GSD, sex is heritable and genetically determined by genetic components such as the sex chromosomes and sex-determining genes [2]. While in species with ESD, sex is controlled by environmental factors such as temperature, pH, hormones, social interaction, and so on [3]. Intriguingly, in many species especially fishes, although the sex has been deduced by genetic factors, the phenotypic sex can be altered under specific environmental conditions [4,5]. Water temperature is one of the major environmental factors that influence the sex of ectothermic fishes. The elevated water temperature may initially arouse the transcriptional changes in sensitive responsory genes, which will subsequently modulate the regulatory pathways for sex determination. Many sex-related genes have been found to alter the expression level under high temperature, thus affecting the sexual phenotype, such as *dmrt1* (doublesex and mab-3 related transcription factor 1), *gsdf* (gonadal soma-derived factor), *cyp19a1a* (gonadal aromatase), *foxl2* (forkhead box l2), etc. [6,7,8,9]. Nevertheless, researchers of the early response factors in the hierarchy and their mechanisms are still limited. How the individual perceives the changes in external temperature, which then affect internal sex differentiation, is still worthy of in-depth investigation.

The eukaryotic translation elongation factor 1 alpha (*eef1a*) gene is well-defined in protein biosynthesis, which plays a vital role in bringing aminoacyl-tRNAs to the ribosome A/P site in a GTP-dependent reaction [10]. There are two isoforms of *eef1a* that have been identified in mammals, *eef1a1* and *eef1a2*. The amino acid sequences translated from these two isoforms have a similarity of over ninety percent. The tissue distribution of these two isoforms is different. *eef1a1* has a broader distribution in multiple tissues except in the muscle and heart, while *eef1a2* expression is restricted in the latter tissues and also certain cells such as motor neurons, islet cells, and enteroendocrine cells [11,12]. It has been found in mammalian cells that eEF1A1, but not the eEF1A2 variant, participated in the heat shock response (HSR) process. Under heat stress, eEF1A1 can trigger the transcription of heat shock protein 70 (HSP70) by recruiting the master regulator heat shock factor 1 (HSF1) to its promoter. It can also contribute to the stabilization of HSP70 mRNA by associating with elongating RNA polymerase II, and facilitating its transportation from the nucleus to the cytoplasm. In addition, severely impaired HSR and compromised thermotolerance were observed in eEF1A1-knocked down cells [13].

Apart from the above role in protein translation and HSR, eEF1A has many other functions including mediating signal transduction and gonad development. In mammals, eEF1A has been shown to directly interact with Phospholipase C-gamma1 on the pleckstrin homology (PH) domain, thus activating phosphoinositol 4-kinase, a member of the signal transduction pathway regulated by growth factors [14]. In Atlantic cod (*Gadus morhua*), the *eef1a1* gene fluctuated during oocyte growth. It was low expressed at the oocyte differentiation stage, and significantly up-regulated through the primary or early secondary growth stage, then gradually down-regulated in the later developmental stage [15]. Recently, eEF1A1 has been found to interact with the *gsdf*, which is essential for testicular differentiation in the adult testis of medaka (*Oryzias latipes*) [16]. The comprehensive role of eEF1A in high-temperature perception, signal transduction, and gonadal development indicated that it may be involved in the perception of external temperature changes and the subsequent sex differentiation regulation.

Chinese tongue sole (*Cynoglossus semilaevis*) is an important economic marine fish that is popularly cultured in coastal areas of China. Our previous study has found a relatively balanced sex ratio in this species under natural conditions, while under high temperature approximately 73% of female fish can be masculinized to form fertile pseudomales [17,18]. The whole genome of *C. semilaevis* was definitively sequenced and the genetic female and male can be distinguished with well-developed simple sequence repeat (SSR) markers [18,19]. Therefore, *C. semilaevis* is a uniquely powerful model to explore the mechanism of high-temperature perception between sexes. However, the responsory genes to temperature in *C. semilaevis* are still unknown. In the present research, the *C. semilaevis eef1a1* gene was identified. Its expression pattern was detected across different tissues and through the gonadal development stage. Moreover, the response of *eef1a1* to environmental high temperature was assessed both in vitro and in vivo. These findings illustrate that *C. semilaevis eef1a1* might have an important role in thermal stress response and gonadal differentiation and development.

## 2. Materials and Methods

### 2.1. Ethics Statement

All work involving *C. semilaevis* was carried out according to the ethical principles of animal welfare of the Yellow Sea Fisheries Research Institute, Chinese Academy of Fishery Sciences (Qingdao, China). All animal experiments were approved by the Institutional Animal Care and Use Committee (IACUC) of YSFRI, CAFS.

### 2.2. Fish Culture, High-Temperature Induction, and Sampling

*C. semilaevis* were purchased from the Haiyang High-Tech Experimental Base (Haiyang, China), and fed two to three times daily with pelleted commercial food of the appropriate size. For expression analysis, normal temperature (22 °C) reared females and males were collected at 30 dpf (day post-fertilization), 50 dpf, 3 mpf (month post-fertilization), 4 mpf, 6 mpf, 9 mpf, 1 ypf (year post-fertilization), 2 ypf and 3 ypf. For high-temperature induction, approximately sixty 30 dpf larvae from the same family were equally divided into two groups and reared in filtered seawater in two 300 L tanks. One group was set as the control, and the larvae were cultivated at 22 °C. The other was the high-temperature treated group, the larvae were cultured at 28 °C until 3 mpf when sex differentiation was completed. For sampling, all fishes were anesthetized with 0.2% tricaine methanesulfonate (MS-222) and then sacrificed. A total of 11 tissues including brain, gill, heart, stomach, liver, spleen, intestine, kidney, muscle, skin, and ovary or testis were sampled from each of three 3 ypf females and males. For fish from 30 dpf to 2 ypf, and for the experiment under high-temperature induction, three individuals of each sex were randomly sampled and the gonads were collected. All samples were immediately transferred to liquid nitrogen and were then stored at −80 °C until RNA extraction. Parts of the caudal fin of each fish were collected and fixed in 100% ethanol.

### 2.3. RNA/DNA Extraction and Sex Determination

Total RNA was isolated from each tissue sample using TRIzol reagent (Invitrogen, Carlsbad, CA, USA) following the manufacturer’s protocol. The quantity and quality of the RNA samples were determined using the NanoDrop 2000 spectrophotometer (Thermo, Waltham, MA, USA). Genomic DNA was extracted from the fins via the phenol-chloroform method. Sex-specific SSR primer pair (Table 1) was used to determine the genetic sex of each fish as previously reported [19].

### 2.4. First-Strand cDNA Synthesis and RACR-PCR

In order to acquire the full-length sequence of *eef1a1*, rapid amplification of cDNA ends (RACE) was performed. Firstly, to obtain the cDNA fragments, reverse transcribed cDNA from purified total RNA was used as a template for the RT-PCR. Then, the RACE-PCR was carried out using the SMARTer RACE 5′/3′ Kit (Clontech, Mountain View, CA, USA) according to the manufacturer’s protocol with gene-specific primers (GSPs) and nested gene-specific primers (NGSPs). PCR primers for the cDNA cloning were listed in Table 1. The purified PCR fragment was subcloned into the pEASY-T1 vector (TransGen, Beijing, China) and sequenced.

### 2.5. Sequence Analysis and Phylogeny of eef1a1

The open reading frame (ORF) of *eef1a1* was deduced by the ORF finder online program (https://www.ncbi.nlm.nih.gov/orffinder/, 4 April 2021), and protein domains were identified by Conserved Domain Architecture Retrieval Tool (CDART) (https://www.ncbi.nlm.nih.gov/Structure/lexington/lexington.cgi?cmd=rps, 4 April 2021). The sequence conservation across species was assessed by multiple sequence alignment of amino acids using DNAMAN software (version 9, Lynnon Biosoft, San Ramon, CA, USA). Orthologous relationship was assessed with the phylogenetic tree of amino acid sequences via the neighbor-joining (NJ) algorithm with 1000 bootstrap replicates using MEGA software (version 11, [20]).

### 2.6. Analysis of mRNA Expression Using Quantitative RT-PCR (qRT-PCR)

The qRT-PCR amplifications were performed with QuantiNova SYBR Green PCR Kit (Qiagen, Hilden, Germany) in an ABI StepOnePlus Real-Time PCR system (Applied Biosystems, Foster City, CA, USA). The first-strand cDNA was synthesized from 1 μg of total RNA using random primers, according to the PrimeScript RT reagent Kit protocol (Takara, Dalian, China). The primers *eef1a1*-qF/qR were designed using the Primer3Plus online software (https://www.primer3plus.com/index.html, 1 May 2021) (Table 1). Each amplification reaction contained 1 μL of a 1:10 dilution of the original cDNA, 10 μL of the 2 × PCR mastermix, 2 μL of ROX Reference Dye, and specific primers in 700 nM in final volume of 20 μL. *β-actin* was employed as the housekeeping reference gene. All reactions were performed in three technical replicates. The cycling condition was 2 min at 95 °C for the initial denaturation followed by 40 cycles at 95 °C and 60 °C for 5 s and 10 s, respectively. Melting curve analysis was performed ranging from 60 to 95 °C to confirm the specific amplification peaks (Figure S1a in Supplementary Materials). The amplification efficiency of each qRT-PCR primer pair was generated using the slopes of the standard curves obtained by 10-fold serial dilutions from 10^0^ to 10^−5^ cDNA templates, then calculated by the formula: efficiency (%) = (10^(−1/slope)^ − 1) × 100 (Figure S1b). The relative change in expression levels of *eef1a1* was analyzed using the comparative Ct method (2^−ΔΔCt^ method).

### 2.7. Promoter Cloning and Plasmid Construction

For *eef1a1* promoter cloning, primer *eef1a1*-P-F/R (Table 1) was designed according to the *C. semilaevis* genomic sequence (NCBI Cse_v1.0) to acquire the 1630 bp region upstream from the start codon (ATG). The *Kpn*I and *Hind*III restriction enzyme sites with a few extra bases were introduced into the forward and reverse primer, respectively. Then the *eef1a1* promoter was cloned and inserted into the *Kpn*I/*Hind*III site of pGL3-Basic (Promega, Madison, WI, USA), a vector that contains the firefly luciferase gene but without a promoter. Positive clones were selected and sequenced to acquire p*eef1a1*-luc recombination plasmid. The pGL-3 Basic vector that has no promoter or enhancer was used as a negative control.

### 2.8. Cell Transfection, Heat Treatment, and Luciferase Assay

Human embryonal kidney (HEK) 293T cell line was maintained in DMEM/F-12 medium (HyClone, Logan, UT, USA) supplemented with 10% fetal bovine serum (FBS) (Gibco, Thorntan, NSW, Australia) and 100 mg/mL antibiotics (penicillin and streptomycin) (Invitrogen, Frederick, MD, USA). Cells were cultured under 5% CO_2_ at 37 °C. Cells were seeded at 2 × 10^5^ cells per well in a 24-well plate. When the confluency reached ~80%, cell transfection was carried out using the Lipofectamine 3000 kit (Invitrogen, Carlsbad, CA, USA). The p*eef1a1*-luc plasmid was added to three wells with the amount of transfected of 500 ng/well, and 40 ng of pRL-TK plasmid was also transfected per well as an internal reference. After 48 h of co-transfection, heat treatment was carried out by gradually increasing the temperature from 37 to 42 °C through water bath heating, and the cells were maintained for an additional 1 h and 2 h, respectively. Then cells were collected, and luciferase activity was calculated as the ratio of firefly luciferase to Renilla luciferase by using the Dual-Luciferase Reporter Assay System (Promega, Madison, WI, USA) using Varioskan Flash (Thermo, Vantaa, Finland). At least three independent experiments were performed under similar experimental conditions.

### 2.9. Data Presentation and Statistical Analysis

Data in this research were presented as means ± S.E.M. Statistical analyses between groups were performed by one-way analysis of variance (ANOVA), followed by Bonferroni’s multiple comparison tests with the GraphPad Prism V6.0 software (GraphPad, San Diego, CA, USA). p value less than 0.05 were considered statistically significant.

## 3. Results

### 3.1. Identification of eef1a1 from C. semilaevis Gonad

**Figure 1:**
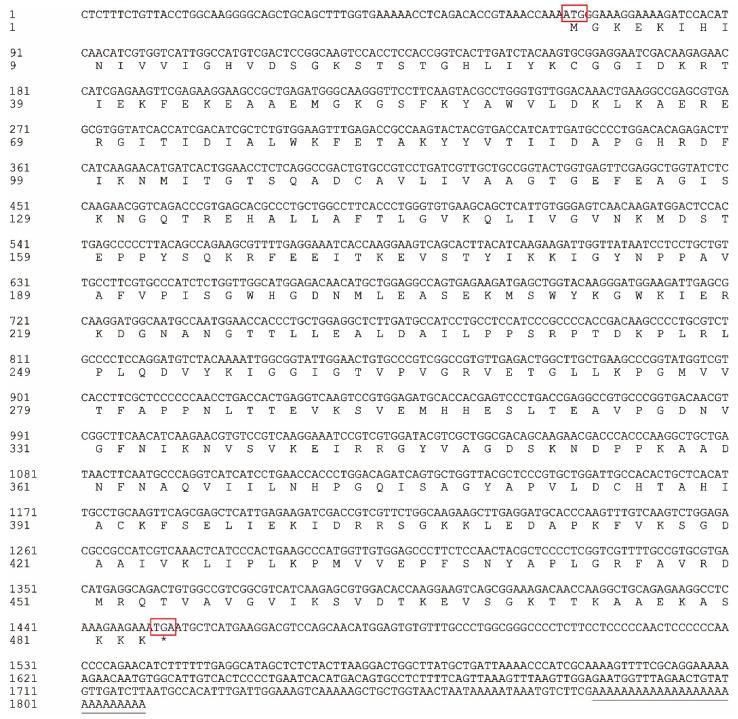
Analysis of nucleotide and deduced amino acid sequence of *C. semilaevis eef1a1*. The initiation codon and termination codon are boxed and colored red. The stop codon is indicated as an asterisk (*). The polyadenylation signal is indicated with a single line.

For identification of *eef1a1*, *C. semilaevis* adult testes were used. After sequencing, the full length of *eef1a1* was 1809 bp, consisting of a 67-bp 5′ untranslated region, a 356-bp 3′ untranslated region, and a 1386-bp ORF (GenBank accession number ON075461) (Figure 1). The deduced protein was 461 amino acids with a calculated molecular weight of 50.40 kDa and a predicted isoelectric point of 9.21. Protein motif analysis identified an EF-1_alpha domain between amino acid residues 1 and 445.

### 3.2. Putative Amino Acid Sequence Comparison and Phylogenetic Analysis

**Figure 2:**
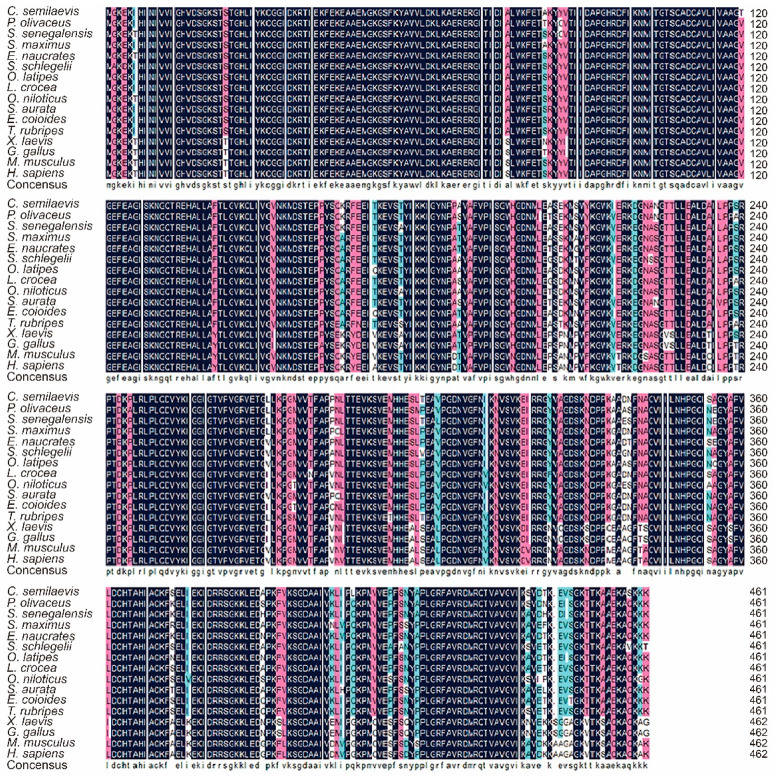
Comparison of *C. semilaevis* eEF1A1 amino acids with those of other vertebrate orthologues. The sequences were identified by BLAST analysis with the following GenBank accession numbers: XP_019960197.1 (*Paralichthys olivaceus*), XP_043908364.1 (*Solea senegalensis*), XP_035476038.1 (*Scophthalmus maximus*), XP_029370759.1 (*Echeneis naucrates*), AGT48259.1 (*Sebastes schlegelii*), NP_001098132.1 (*Oryzias latipes*), XP_019109285.2 (*Larimichthys crocea*), NP_001266576.1 (*Oreochromis niloticus*), XP_030267850.1 (*Sparus aurata*), AOW69105.1 (*Epinephelus coioides*), NP_001032962.1 (*Takifugu rubripes*), XP_018092534.1 (*Xenopus laevis*), NP_001027570.3 (*Gallus gallus*), NP_034236.2 (*Mus musculus*), AAK95378.1 (*Homo sapiens*). Similar amino acids were shaded by box shade. Purple boxes represented identical residues, pink boxes represented > 75% homology residues, and blue boxes represented >50% homology residues.

**Figure 3:**
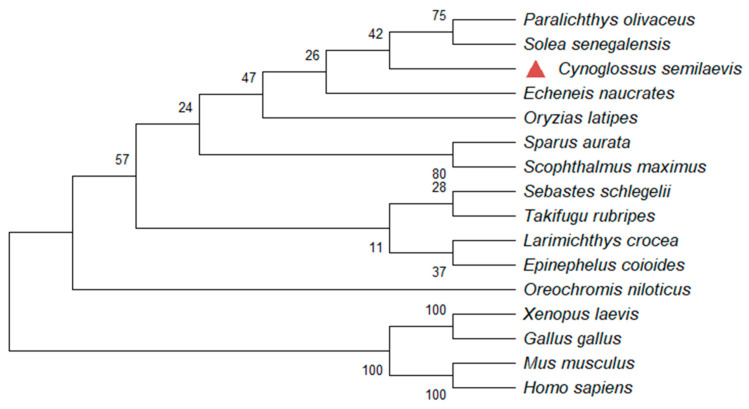
Phylogenetic relationships of *C. semilaevis* eEF1A1 from other representative species. The Neighbor-Joining tree was constructed by MEGA-11 1000 bootstrap replicates based on the full-length amino acid sequences same as above. *C. semilaevis* eEF1A1 was highlighted with red triangle. The relative genetic distances were explained by the scale bar and the branch lengths.

The deduced amino acid sequence of the *C. semilaevis* eEF1A1 was aligned with other homologues, as shown in Figure 2. The eEF1A1 exhibited an extremely high identity from 84.35% with eEF1A1 of *Gallus gallus* to 94.13% with eEF1A1 of *Oryzias latipes* (Figure 2). To further analyze the evolutionary relationship of *C. semilaevis* eEF1A1 with other homologues, the NJ phylogenetic tree was constructed and a clear division was observed between teleosts and other vertebrates including amphibians, birds, and mammals (Figure 3). The molecular relationship indicated by this tree was consistent with the taxonomic classification of these species.

### 3.3. Spatial Distribution of eef1a1 in Adult C. semilaevis

**Figure 4:**
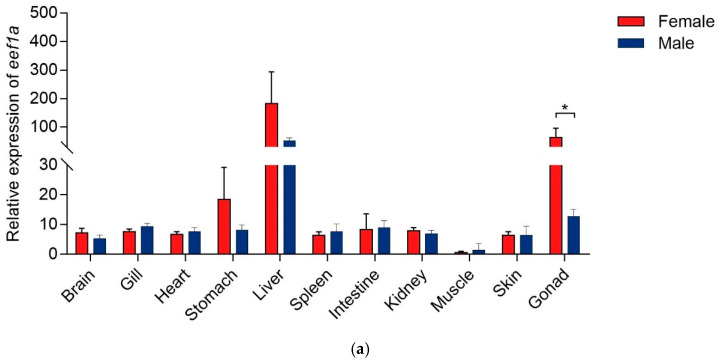

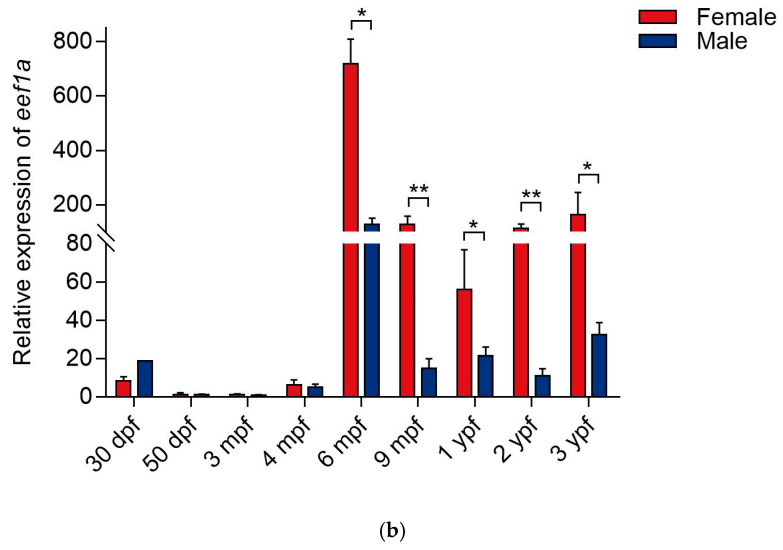
Expression pattern of *eef1a1* in *C. semilaevis*: (**a**) Spatial distribution of *eef1a1* in 3 ypf adult *C. semilaevis* maintained at normal temperature (22 °C). (**b**) Relative expression level of *eef1a1* at different stages of the ovary and testis in *C. semilaevis* maintained at normal temperature. The expression levels were presented as mean ± S.E.M. (*n* = 3). Asterisks represented statistically significant difference (*, *p* < 0.05; **, *p* < 0.01).

For qRT-PCR, the amplification specificity was confirmed by melting curve analysis, and similar efficiency levels were further observed between *eef1a1*-qF/R (107.66%) and *β-actin*-qF/R (105.78%) (Figure S1). The spatial expression profile in 3 ypf adult *C. semilaevis* maintained at normal temperature (22 °C) indicated that *eef1a1* could be detected in many analyzed tissues, including liver, gonad, stomach, brain, gill, heart, spleen, intestine, and skin. *eef1a1* exhibited higher expression level in liver than in other tissues, and it can hardly be detected in the muscle. Intriguingly, *eef1a1* showed a sexually dimorphic expression pattern in the gonads with a statistically higher expression level in the ovary than in the testis (Figure 4a).

### 3.4. Temporal Expression of eef1a1 during Development of Gonad

We further investigated the dynamic changes in *C. semilaevis eef1a1* during the development of gonads from normal temperature (22 °C) reared fish. Overall, the expression level of *eef1a1* in females and males was relatively low from 30 dpf to 4 mpf, increased dramatically at 6 mpf, then decreased at 9 mpf, and kept a stable level till 3 ypf. In particular, the expression levels of *eef1a1* in the ovary were significantly higher than those in the testis from 6 mpf to 3 ypf (*p* < 0.05) with a ratio from 10.25 to 2.59 times (Figure 4b).

### 3.5. Expression Pattern of C. semilaevis eef1a1 under High-Temperature Induction

**Figure 5:**
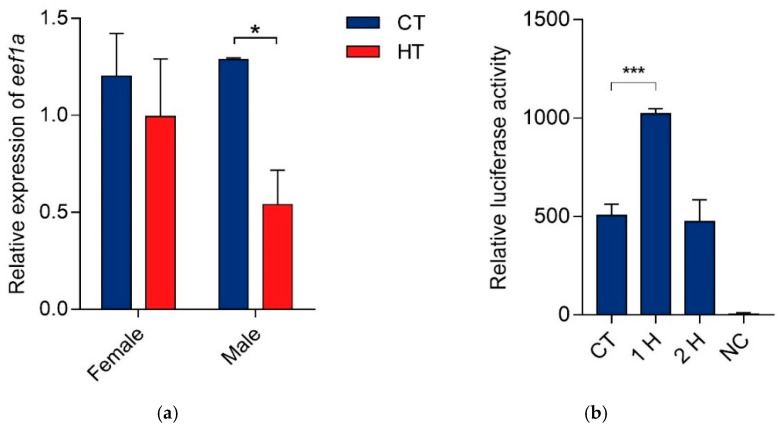
*eef1a1* expression patterns under high temperature: (**a**) Gonadal *eef1a1* expression patterns in *C. semilaevis* cultured under high temperature and normal temperature from 30 dpf to 3 mpf. Relative expression levels at 3 mpf were presented as mean ± S.E.M. (*n* = 3). CT: control temperature (22 °C); HT: high temperature (28 °C). (**b**) *C. semilaevis eef1a1* promoter activity analysis under high temperature. Luciferase activities were shown as mean ± SEM (*n* = 3). CT: control temperature (37 °C); 1 H: high temperature (42 °C) treated for 1 h; 2 H: high temperature treated for 2 h; NC: *pGL3-Basic* as negative control. Asterisks represented statistically significant difference (*, *p* < 0.05; ***, *p* < 0.001).

To evaluate the potential role of *eef1a1* during *C. semilaevis* sex differentiation, the *eef1a1* expression was detected in females and males treated with high temperature (28 °C) from 30 dpf to 3 mpf. After the induction experiment, gonads from 3 mpf fish were sampled for qRT-PCR. Compared to fishes cultured under normal temperature (22 °C), *eef1a1* was statistically down-regulated in males treated with high temperature, while the difference was not detected in females (Figure 5a). The different performance between sexes indicated that *eef1a1* may participate in the high temperature-induced sex reversal in *C. semilaevis*.

### 3.6. eef1a1 Can Response to High Temperature Rapidly

To evaluate if *eef1a1* can respond to environmental high temperature, the *eef1a1* promoter sequence of 1630 bp upstream from the start codon was cloned for activity analysis. The 293T cell was then transfected with *peef1a1-luc* reconstructed vector. After high temperature (42 °C) treatment for 1 h, the *eef1a1* promoter activity increased rapidly to 2.028 times that of the control group (*p* < 0.05), and then returned to the nearly normal level after 2 h treatment (Figure 5b). These results suggested that *eef1a1* can respond rapidly to high temperature.

## 4. Discussion

In the present study, the *eef1a1* gene from *C. semilaevis* was isolated and its complete sequence was characterized. The deduced eEF1A1 protein consisted of 461 amino acids and possessed a conserved EF-1_alpha domain. The extremely high identity between the *C. semilaevis* eEF1A1 and homologues from other species indicated the structural conservation of this polypeptide during vertebrate evolution, which may be owed to its essential role in the core biological process of protein translation.

Based on the expression analyses, *eef1a1* was highly abundant and exhibited a wide distribution in different tissues in adult *C. semilaevis*. This tissue distribution pattern was consistent with *eef1a1* in other teleosts and mammals [21,22,23], which further supported that the transcript cloned in this study was the definite isoform. It is worth noting that *eef1a1* was expressed relatively high in the gonad, with a statistical female-biased sexual dimorphism. The expression level of *eef1a1* peaked at 6 mpf, and remained high in the adult ovary. Previous research has reported that *C. semilaevis* finished gonadal differentiation and went on to develop at 6 mpf, which was followed by an important period for ovum development and yolk energy reserve [24]. Thus, this expression pattern of *eef1a1* may be related to its role in protein synthesis, and *eef1a1* may play a vital role during gonadal development.

The expression of *eef1a1* was relatively low from 30 dpf to 4 mpf in the gonad, with no statistical difference between female and male. The ovary and testis differentiation in *C. semilaevis* can be distinguished at around 60 dpf under the histological observation, and cellular differentiation can be detected at 4–5 mpf [25]. To further confirm if *eef1a1* was involved in sex differentiation, we treated *C. semilaevis* with a high temperature (28 °C) from 30 dpf to 3 mpf, which was the temperature-sensitive period of sex determination [17,18,26]. After the long-term heat treatment, we found significant down-regulation of *eef1a1* specifically in the testes of males. A possible explanation is that males cannot undergo sex reversal, thus shutting down their sensory pathway as a consequence of the adaptive mechanism. While in females, who possess the switchable sex-determining pathways, the ability of sensing high temperature needs to be maintained during the critical period. Therefore, *eef1a1* may keep a high expression level as a guarantee for females perceiving the environmental signals and, thus, undergo sex reversal.

The heat response ability of *eef1a1* was also tested in the in vitro analysis and the result showed that *eef1a1* can respond to high temperature rapidly. Under the promoter activity of *eef1a1*, the *luciferase* reporter gene exhibited a significant elevation at 1 h after heat stress, which descended to a normal level at 2 h heat exposure. It has been reported in mammals that eEF1A1 rapidly activates transcription of HSP70 upon heat stress from 0.5 to 1 h by recruiting HSF1 to its promoter and ends at 2 h of heat shock. *eef1a1* knock-down cells exhibit severely impaired HSR and compromised thermotolerance [13]. Thus, the rapid and efficient transcription feature of *eef1a1* might be conserved in vertebrates, from teleosts to mammals.

Taken together, the present study provided fundamental information indicating that *C. semilaevis eef1a1* might have a dual role in the perception of external temperature changes as well as sex differentiation and gonadal development. Nevertheless, the difficulty in pseudomale identification ahead of sex determination and lacking fish cell lines have hindered the research of *eef1a1* in sex reversal. Solutions to these questions will enhance the understanding of the role that *eef1a1* plays in the molecular network underlying temperature-dependent sex reversal.

## 5. Conclusions

In conclusion, the 1809 bp *eef1a1* gene was identified and analyzed in *C. semilaevis*, a species whose sex can be influenced by environmental temperature. The *eef1a1* was highly conserved with homologues from other species, and expressed widely across tissues except for the muscle. In the gonad, *eef1a1* expressed consistently higher in the ovaries than that in testes from 6 mpf to 3 ypf, with a peak at 6 mpf, indicating its potential role in gonad development. In addition, in *C. semilaevis* treated with high temperature (28 °C) during the temperature-sensitive period of sex determination, *eef1a1* specifically down-regulated in the testes while keeping its expression level in the ovary. In vitro analysis further found that *eef1a1* can respond to high temperatures rapidly. These results suggest that *C. semilaevis* *eef1a1* might be a candidate response-related gene that can link thermal stress perception and gonadal differentiation and development.

## Figures and Tables

**Table 1:** Primers used in this study.

Primer Name	Sequences (5′–3′)	Purpose
*eef1a1*-cds-F	ATGGGAAAGGAAAAGATCCACATCA	Partial fragment amplification
*eef1a1*-cds-R	TCATTTCTTCTTTGAGGCCTTCTCT	
*eef1a1*-5′GSP	AAGTGACCGGTGGAGGTGGACTTGC	5′RACE
*eef1a1*-5′NGSP	TTTTGGTTTACGGTGTCTGAGGT	
*eef1a1*-3′GSP	TTGTCAAGTCTGGAGACGCCGCCAT	3′RACE
*eef1a1*-3′NGSP	CTGTGGCCGTCGGCGTCATCAA	
*eef1a1*-P-F	ggggtaccTCACAGCACAGT	Promoter amplification ^1^
*eef1a1*-P-R	cccaagcttTTTGGTTTACTGAATAAAAAGAAAAGAA	
*eef1a1*-qF	ACTTCAATGCCCAGGTCATC	qRT-PCR
*eef1a1*-qR	AACTTGCAGGCAATGTGAGC	
*β-actin*-qF	GCTGTGCTGTCCCTGTA	qRT-PCR
*β-actin*-qR	GAGTAGCCACGCTCTGTC	
sex-F	CCTAAATGATGGATGTAGATTCTGTC	Genetic sex identification
sex-R	GATCCAGAGAAAATAAACCCAGG	

^1^ Uppercase letters indicate the primer sequence and lowercase letters indicate the restriction enzyme sites with protective nucleotides.

## Data Availability

No new data were created or analyzed in this study. Data sharing is not applicable to this article.

## Supplementary Materials

The following are available online at https://www.mdpi.com/article/10.3390/ani12131603/s1, Figure S1: Efficiency and Melting curve analysis of primers used for qRT-PCR: (**a**) Melting curve analysis of qRT-PCR primer sets in *C. semilaevis*. (**b**) Amplification of 10-fold serial dilutions ranging from 10^0^ to 10^5^ of the cDNA templates for efficiency calculation.
