# Supplementary material for: A Potential Role of the Translation Elongation Factor eef1a1 in Gonadal High-Temperature Perception in Chinese Tongue Sole (Cynoglossus semilaevis)
Source: Animals (Basel). 2022 Jun 21;12(13):1603. doi: 10.3390/ani12131603 (PMC9265046; doi:10.3390/ani12131603)
Supplement: Supplementary file 1 [file animals-12-01603-s001.zip › animals-1683438-supplementary.pdf]

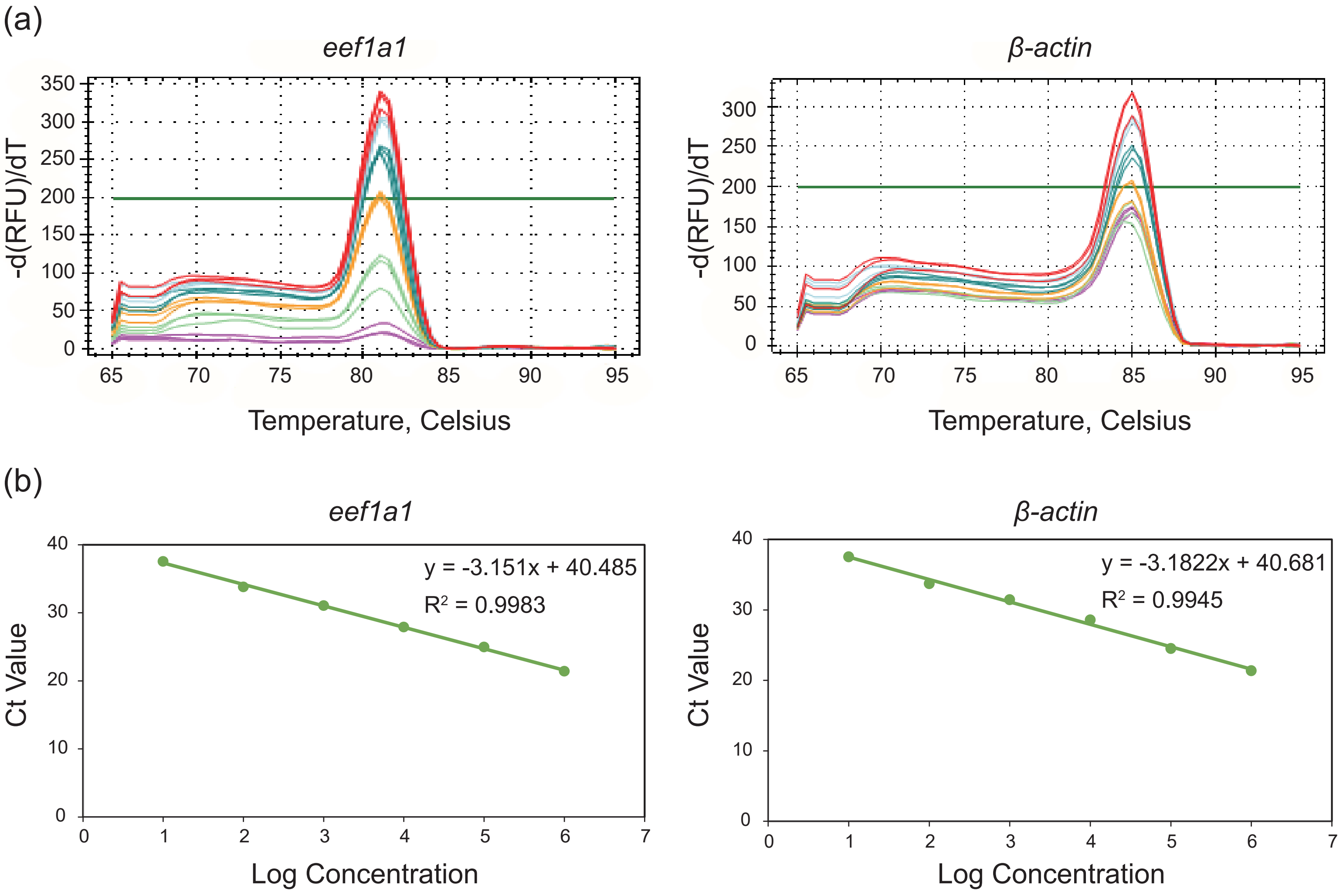

Figure S1. Efficiency and Melting curve analysis of primers used for qRT-PCR: (**a**) Melting curve analysis of qRT-PCR primer sets in C. semilaevis. (**b**) Amplification of 10-fold serial dilutions ranging from 100 to 105 of the cDNA templates for efficiency calculation.
